# Morphology and Immunoexpression of Selenoproteins in Term Placenta of Alpaca (*Vicugna pacos*) from the Peruvian Andes

**DOI:** 10.3390/biology14010064

**Published:** 2025-01-14

**Authors:** Miluska Beatriz Navarrete Zamora, Francisco Acuña, Mônica Duarte da Silva, Thamires Santos-Silva, Matheus Henrique Herminio Garcia, Rodrigo da Silva Nunes Barreto, Alberto Sato Sato, Claudio Gustavo Barbeito, Maria Angelica Miglino

**Affiliations:** 1 Facultad de Medicina Veterinaria, Universidad Nacional Mayor de San Marcos, Lima 15001, Peru; mnavarretez@unmsm.edu.pe (M.B.N.Z.);; 2 Facultad de Ciencias Veterinarias, Universidad Nacional de La Plata, La Plata 1900, Buenos Aires, Argentina; barbeito@fcv.unlp.edu.ar; 3 Department of Surgery, School of Veterinary Medicine and Animal Science, University of São Paulo, São Paulo 05508-270, São Paulo, Brazil; monicasilva@usp.br (M.D.d.S.); thamiresssilva@usp.br (T.S.-S.); rodrigobarreto@usp.br (R.d.S.N.B.); 4 Department of Animal Anatomy, University of Marilia, Marília 17525-902, São Paulo, Brazil; matheushenrigarcia@gmail.com (M.H.H.G.); miglino@usp.br (M.A.M.)

**Keywords:** camelids, extreme environments, fetus, selenium, placenta

## Abstract

South American camelids inhabit high-altitude environments characterized by hypoxia, influencing embryonic, fetal, and placental development. This study examined the term placenta morphology of alpacas (*Vicugna pacos*, N = 12) and the immunoexpression of antioxidant selenoproteins (SP). We hypothesize that the placenta of alpacas, adapted to high altitudes, has characteristics with other species also adapted to altitude. Placentas were paraffin-embedded, sectioned (3–5 µm), stained with hematoxylin–eosin (H&E), Masson’s trichrome, and picrosirius red, and analyzed via light and polarized light microscopy. The chorion showed simple cuboidal epithelium with binucleated cells, a subepithelial mesenchyme rich in blood capillaries (area: 124.90 ± 9.82 µm^2^), and type III collagen fibers. The chorionic villi measured 2740.22 ± 132.75 µm. The allantois contained a simple columnar epithelium and mesenchyme with type I collagen fibers. Immunohistochemistry localized SP-N, SP-P, Dio-3, and GPx-3 in the blood capillaries and mesenchymal tissue of the chorion but not in the allantois. These findings were compared to human and sheep placentas from different altitudes due to a lack of camelid data at low levels. The morphological features resembled adaptations to hypoxia observed in other species. This preliminary study suggests a potential role for selenoproteins in hypoxia adaptation, providing a basis for future functional studies.

## Simple Summary

South American camelids are valuable economic resources, and understanding various aspects of each species’ biology ensures their care and sustainable use. Little is known about the reproduction of the alpaca (*Vicugna pacos*) under high-altitude conditions and the influence of different factors, such as low oxygen availability, on gestation. Low oxygen levels (hypoxia) modulate embryonic–placental development. This study examined the morphology and expression of antioxidant molecules (selenoproteins) in the placenta of the alpaca, a native of the Peruvian Andes. The results were compared with studies conducted in other species (humans and sheep). In the chorion, abundant subepithelial capillaries were observed, showing markers for the studied selenoproteins. Conversely, no markers for any selenoproteins were found in the amnion. Additionally, the area of the capillaries and the length of the chorionic villi were measured. The findings in alpacas suggest adaptations of the placenta to hypoxic conditions. Future comparative studies with alpacas that live and reproduce at low altitudes will be necessary to further explore the findings obtained.

## 1. Introduction

High-altitude environments (HAEs), as an abiotic factor, drive adaptations in endemic species inhabiting these areas [1]. Among the mammals living in HAEs are South American domestic camelids (SACs). Among the SACs, there are wild species such as the guanaco (*Lama guanicoe*) and the vicuña (*Vicugna vicugna*), as well as domesticated ones like the llama (*Lama glama*) and the alpaca (*Vicugna pacos*) [2,3]. SACs are an economically significant resource, as they have been bred for meat, milk, and fiber production by Andean communities for centuries [4]. SACs have morphological and physiological adaptations that enable them to inhabit and survive in HAEs characterized by low oxygen availability, rocky substrates, and low temperatures. Some of these adaptations include soft plantar pads, thick insulating fiber, a high hemoglobin–oxygen affinity, and small elliptical red blood cells with high hemoglobin concentrations [2,5]. On a reproductive level, adaptation to HAEs has improved fetal growth restriction in mammals studied to date, including guinea pigs, high-altitude sheep [6], and human populations such as Andeans and Tibetans [7,8]. Specifically in SACs, some morphological data on the placenta suggest potential adaptations to the characteristics of the Andean HAE [9,10].

The placenta of SACs is diffuse and epitheliochorial [9,10,11,12,13], showing similarities with phylogenetically related species such as Old World camelids [14,15,16], the sow [17,18], the mare [19,20,21], and the ewe [22,23]. In the term placenta of the SAC *Vicugna pacos*, a minimal interhemal distance has been observed compared to the epitheliochorial placentas of other domestic ungulates. This feature may represent an adaptation of pregnancy to high altitudes [9,10]. Studies in other ungulates, such as the ewe, have found that the placenta of females living at high altitudes (HAs) has a larger maternal–fetal contact surface, which would ensure substance exchange [24]. Comparisons between pregnant ewes maintained at different altitudes showed that the total surface area of cotyledons and the area occupied by vasculature were greater in females living at HA than in those at low altitudes (LAs) [25]. In addition to studies on ungulates, analyses were also conducted on the placenta of humans living at different altitudes. In these cases, the placenta is of the hemochorial type, meaning the chorionic villi are in direct contact with maternal blood [8]. In the hemochorial placenta of humans living at HA, variations in the arrangement of chorionic villi have been observed compared to pregnant women at LA. The differences in the branching patterns of the chorionic villi between the two groups could be due to increased angiogenesis stimulated by the lower partial pressure of oxygen prevailing at high altitudes [26]. It could even be thought that the modifications could be due to the effect of hypoxia on different tissue components. For example, collagen production is regulated by hypoxia [27]. Subsequent studies demonstrated that human placentas from women living at HA also show increased vascularization of the villi, thinning of the villous membranes, proliferation of the villous cytotrophoblast, and reduced perivillous fibrin deposition compared to the placentas of women at LA [28]. Andean women have on average higher uterine blood flow and thus a greater oxygen supply during pregnancy [29]. The hypoxic conditions present in HAEs induce oxidative stress and reduce the antioxidant capacity of certain molecules, leading to placental insufficiencies [30,31]. Some molecules involved in preventing cellular damage from oxidative stress contain the mineral selenium (Se) and are known as selenoproteins. Examples of selenoproteins (SPs) include SP-P, SP-N, glutathione peroxidase 3 (GPx-3), and iodothyronine deiodinase 3 (Dio-3). These selenoproteins also play a role in transporting Se to intracellular compartments via transmembrane receptors [32]. The effect of Se during pregnancy and its relationship with the development of the embryo and placenta has been studied in both humans and mice. Studies on pregnant humans and mice under Se-deficient conditions have shown that the placental tissue develops compensatory mechanisms to sufficiently supply this mineral to the fetus [33,34]. It has been suggested that the Se concentration in amniotic and allantoic fluids could serve as an indicator of fetal Se status throughout gestation [35].

Andean women exhibit greater antioxidant capacity and reduced oxidative stress during pregnancy compared to European residents at high altitude [36]. In SACs, there are no studies specifically addressing the role of Se and selenoproteins in placentation and the placenta, unlike studies conducted on blood. A comparison of serum Se levels in llamas and alpacas showed no significant variations across the late pregnancy, peripartum, and late lactation stages [37,38]. Similarly, in other ungulates, such as goats, no differences in serum Se levels were found among females at different gestational stages [39]. These findings highlight that environmental conditions, such as hypoxia, influence placentation and consequently embryonic/fetal development. Understanding the morphofunctional characteristics of the placenta in species adapted to high altitudes, such as SACs, is essential for comprehending gestational complications. In terms of reproductive efficiency, camelids exhibit a low performance, with an estimated success rate of only 50% in SACs [40,41]. Furthermore, the annual abortion and stillbirth rate is approximately 10% [42]. In such cases, it is crucial to evaluate the placenta for potential anomalies, including incomplete development, placentitis, edema, or other abnormalities [43,44,45]. We hypothesize that the placenta of alpacas, adapted to high altitudes, has characteristics with other species also adapted to altitude. Also, we propose that selenoproteins may protect against reactive oxygen species (ROS) generated under hypoxic conditions.

This study focuses on the morphology and immunoexpression of selenoproteins in the term placenta of alpacas from the Peruvian Andes.

## 2. Materials and Methods

### 2.1. Animals

**Figure 1:**
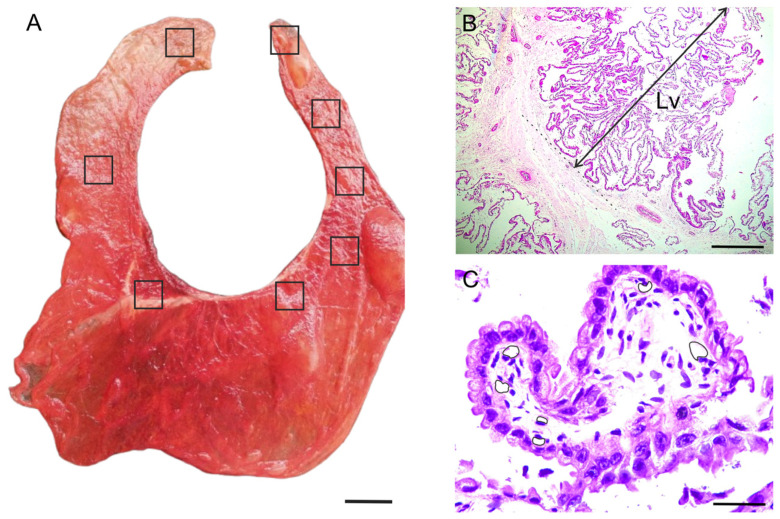
(**A**) Macroscopic view of the term placenta of the alpaca *Vicugna pacos*. The boxes indicate the sites from which the samples were taken. (**B**,**C**) Morphometric variables measured in the chorion. (**B**) Lv, length of the primary villus. (**C**) Area of the blood capillaries (each line delineates the area of each blood capillary). Scale bar: 5 cm (**A**), 500 µm (**B**), 50 µm (**C**).

For the present study, twelve (n = 12) alpaca term placentas were collected in Cusco, Peru, in the Peruvian Andes (altitude = 4338 m), from apparently healthy females that gave birth to healthy offspring after a full-term pregnancy. In alpacas with uncomplicated pregnancies, the placentas appeared reddish-burgundy in color and had a velvety texture [12]. In this study, we used these macroscopic characteristics to identify healthy term placentas (Figure 1A). The twelve placentas were collected within 24 h after delivery. This study was approved by the animal ethics committees of the Faculty of Veterinary Medicine and Animal Science of the University of São Paulo (n° 7213120719).

### 2.2. Sampling and Histological Techniques and Morphometry

The placentas were fixed in 10% paraformaldehyde for histological and immunohistochemical analyses. Random samples consisting of eight sections from each of twelve placentas were collected to examine the chorion and allantois. Three samples were collected from the chorionic sac of the non-pregnant horn: one from the blind end, one from the middle, and one near the bifurcation. In the case of the pregnant horn, five samples were collected: one from the blind end, one from the bifurcation area, one from the center, and two others from the midpoint between the center and the ends (Figure 1A). The samples were then dehydrated in a progressive series of ethanol concentrations (70% to 100%). Subsequently, the samples were cleared in xylene (I and II) and embedded in paraffin. Sections were cut at 5 µm using a microtome (LEICA RM2065, Los Angeles, CA, USA) and stained with hematoxylin and eosin (H&E) for histological examination, Masson’s trichrome to visualize the collagen fibers, and picrosirius red to assess collagen maturation. The slides were analyzed under a light microscope (FV1000 Olympus IX91, Tokyo, Japan) and a polarized light microscope (Olympus BX60, Los Angeles, CA, USA) at the Advanced Image Diagnostic Center (CADI-FMVZ/USP).

The sections stained with H&E were used for morphometric studies. Microphotographs of five fields from the chorion of each female were captured to measure the variables, the length of the primary villus (Lv; linear measurement from the base to the apex) and the area of the blood capillaries, using ImageJ software (https://imagej.net/ij/, accessed on 16 September 2024) (Figure 1B,C). Mean values and deviations for each variable were calculated using InfoStat software (https://www.infostat.com.ar/, accessed on 16 September 2024).

### 2.3. Immunohistochemistry

Sections of the samples were placed on positively charged slides, deparaffinized using a xylene substitute (Neo-Clear^®^), and rehydrated in 100° ethanol. Endogenous peroxidase activity was blocked by incubating the sections in methanol containing 3% hydrogen peroxide. The sections were then rehydrated in decreasing concentrations of ethanol (96% to 50%) and washed in phosphate-buffered saline. For antigen retrieval, the slides were immersed in citrate buffer with hydrochloric acid, heated in a microwave at 750 watts, and then cooled to room temperature. The blocking of nonspecific binding was performed with 1% bovine serum albumin at room temperature. Subsequently, the slides were incubated with the primary antibody overnight at 4 °C, followed by incubation with the secondary antibody at room temperature. Signal amplification was performed with avidin and biotinylated horseradish peroxidase (AB reagents, Santa Cruz Biotechnology, Inc.^®^ laboratory kit, Paso Robles, CA, USA). The staining was developed using 3,3′-diaminobenzidine tetrahydrochloridediaminobenzidine (DAB) and examined under a microscope. Counterstaining was performed with Harris hematoxylin, followed by dehydration in increasing concentrations of ethanol and a xylene substitute (Neo-Clear^®^) [46]. The primary antibodies used, their standardized dilutions, and positive control are detailed in Table 1. A negative control was established by incubating the samples with immunoglobulin G (Vector Laboratories BA1000). Image analysis was performed using a microscope (Leica DM750, Wetzlar, Germany) equipped with an integrated digital camera (ICC50W and Leica Microsystems LAS 4.12 software). Labeling intensity was classified on a semi-quantitative scale (negative, weak, moderate, or strong) and described based on the digital images [47].

## 3. Results

### 3.1. Histology of the Chorion and Allantois of the Alpaca Placenta

**Figure 2:**
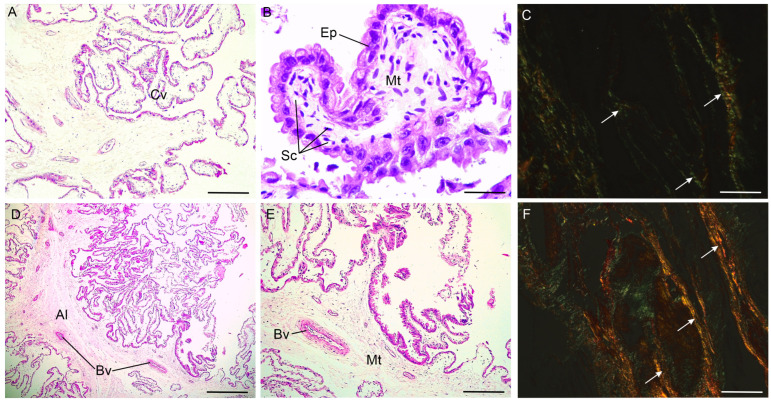
Histological characteristics of the chorion (**A**–**C**) and allantois (**D**–**F**) of the term placenta of the alpaca *Vicugna pacos*. (**A**) Chorionic villi (Cv). (**B**) Details of the epithelium (Ep), mesenchymal tissue (Mt), and subendothelial capillaries (Sc) of the chorionic villi. (**C**) Predominance of type III collagen fibers (arrows). (**D**) Allantois (Al). Blood vessels (Bv) with marked acidophilia in the tunica media are observed. (**E**) Greater detail of the mesenchymal tissue (Tm) and blood vessels (Bv) of the allantois. (**F**) Predominance of type I collagen fibers (arrows). Scale bar: 500 µm (**D**), 200 µm (**A**,**E**), 50 µm (**B**,**C**,**F**).

The average length of the chorionic villi was 2740.22 ± 132.75 µm. In the chorionic villi, the trophoblastic epithelium is composed of simple cuboidal cells. Additionally, binucleated cells are interspersed among the cells along the epithelium. In the mesenchymal tissue of the chorion, blood vessels with different diameters were observed. Numerous blood capillaries underlying the epithelium were found (Figure 2A,B). These blood capillaries had an average area of 124.90 ± 9.82 µm^2^. Regarding collagen distribution in the chorion, the layer in direct contact with the uterus is particularly rich in thin fibers of collagen, predominantly type III collagen (Figure 2C).

Attached to the chorion is the allantois. This adnexa has a mesenchymal connective tissue with fibroblasts, abundant collagen fibers, and larger blood vessels with a marked acidophilia (Figure 2D). It also has a layer of columnar cells arranged in a folded pattern, with eosinophilic cytoplasm and basophilic basal nuclei (Figure 2E). Unlike the chorion, the connective tissue of the allantois is rich in thicker collagen fibers, mainly type I collagen. These fibers form bundles (Figure 2F).

### 3.2. Immunoexpression of Selenoproteins

**Figure 3:**
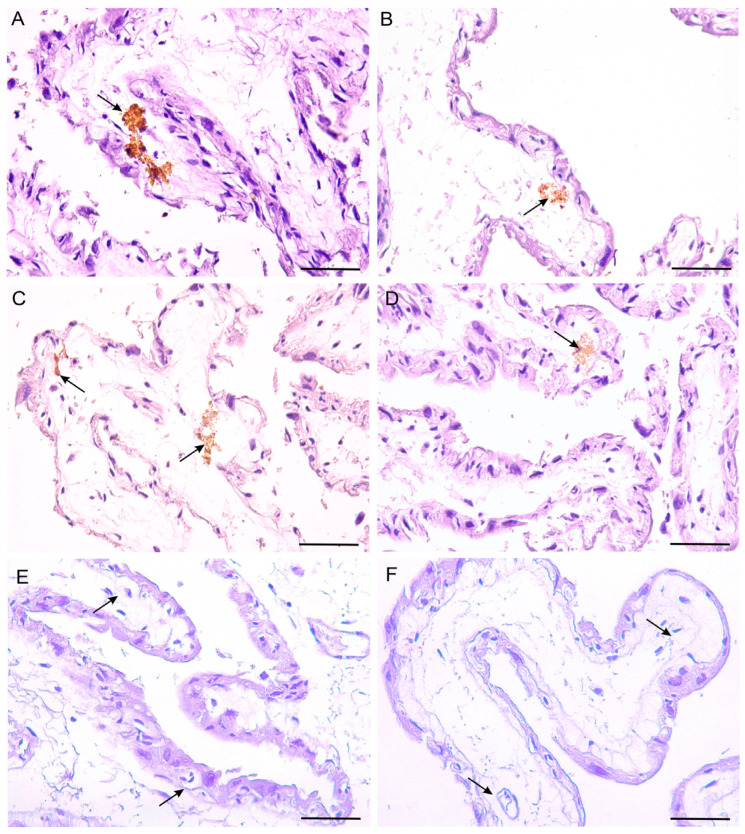
Immunolocalization of selenoproteins SP-P, SP-N, Dio-3, and GPx-3 in the chorion of the term placenta of the alpaca *Vicugna pacos*. The arrows indicate blood capillaries. (**A**,**B**) Strong intensity for selenoproteins SP-P and SP-N, respectively. (**C**,**D**) Moderate intensity for selenoproteins Dio-3 and GPx-3, respectively. (**E**,**F**) Negative controls for selenoproteins SP-P (**E**) and GPx-3 (**F**). Scale bar: 100 µm.

The immunolocalization for each of the four selenoproteins was positive and specifically found in some fetal blood capillaries adjacent to the epithelium of the extreme of the chorionic villi. Additionally, immunostaining was observed inside the blood capillaries and also, outside them, in the mesenchymal tissue. The appearance of the immunostaining was granular, similar to that observed in the positive controls for each selenoprotein. The intensity of the staining was assessed semi-quantitatively. The intensity of the immunostaining was strong for SP-P (Figure 3A) and SP-N (Figure 3B), while it was moderate for Dio3 (Figure 3C) and GPx3 (Figure 3D). No immunostaining in the negative control was observed (Figure 3E,F). In the allatoid, no immunostaining was observed for any of the four selenoproteins.

## 4. Discussion

### 4.1. Adaptation of the Placenta to High Altitude

Animals inhabiting the high altitudes of Peru exhibit greater morphological and functional adaptations in the placenta due to adverse environmental conditions such as the limited availability of resources, including food and oxygen [9,10,48]. To compensate for the low availability of oxygen and nutrients, the placental subepithelial capillary network is enlarged, with increased pro-angiogenic stimulation leading to neovascularization [42,49]. The low-oxygen environment also facilitates proper placental detachment, minimizing the risk of blood loss [50]. This adaptability is further observed in the timing of alpaca births, which occurs in the early morning hours between 6 am and 9 am, when temperatures are less extreme [28,51,52,53].

The epitheliochorial placenta of the alpaca, like that of the pig, the mare, and cetaceans, has structural modifications, commonly called areolas or microcotyledons [54,55,56,57]. In alpacas, these modifications favor the molecular exchange between maternal and fetal cells, providing a reservoir of nutrients to the fetus [10,49]. In relation to this exchange, our study observed capillaries beneath the chorionic epithelium, along with other blood vessels with larger diameters and pronounced acidophilia in the tunica media. Studies on the placentas of Andean women living at high altitudes (HAs) have identified numerous fetal capillaries in the chorionic villi compared to women residing at low altitudes (LAs) [58]. Additionally, Andean women at HA, unlike their LA counterparts, showed more numerous and longer capillaries [59]. These observations were also noted in studies conducted on sheep [24]. It appears that, regardless of placental type, the effects of HA on placental morphology exhibit similarities, particularly concerning fetal blood capillaries. Some authors have suggested that the increased number of blood capillaries in chorionic villi is attributable to enhanced capillary branching under hypoxic conditions [60]. In this context, the abundance of blood capillaries found in the alpaca chorion may represent an adaptation of the placenta to the hypoxic conditions of Andean HAs. However, we did not find reports of studies conducted on camelids at low altitudes to allow for comparisons and the further discussion of our results.

### 4.2. Placental Morphology of Alpacas Living at High Altitudes in the Peruvian Andes

In the present study, two variables (villus length and blood capillary area) were quantified in the chorion of the alpaca. Although these results represent the first such reports, we believe their interpretation requires comparison with values obtained from the chorionic villi of alpacas living at lower altitudes to determine whether hypoxic conditions influence these structures. Morphometric studies have determined that the volume of the trophoblast and the connective tissue underlying the chorion is reduced in the chorionic villi of women living under hypoxic conditions [61]. In our study, we observed variations in the fibrillar components of the connective tissue of the chorion and the allantois. Type III collagen was more abundant in the chorion, while type I collagen predominated in the allantois. Collagen type III fibers provide support and resistance, whereas collagen type I fibers contribute to structural connectivity [62]. There is no direct report on the relationship between the hypoxic microenvironment and collagen expression at the maternal–fetal interface. However, it has been reported that collagen production is regulated by hypoxia in other tissues and cell types [27]. In this context, the abundance of both collagen types may be modulated by the hypoxic conditions of the Peruvian Andes, contributing to support and connectivity in both components of the alpaca placenta. This hypothesis could be tested through comparative and morphometric studies of the extra-embryonic tissues of alpacas living at different altitudes. The proximity of the fetal capillaries to the trophoblastic epithelium significantly impacts nutrient transportation. In alpacas, trophoblastic stretching, which becomes more evident in late pregnancy, facilitates the transfer of nutrients from the maternal to the fetal bloodstream [63,64].

### 4.3. Potential Role of Selenoproteins in the Pregnancy of Alpacas at High Altitudes

The transfer of substances across the maternal–fetal barrier depends on its thickness and extent, the concentration gradient, and the presence of active transport mechanisms [65]. Unlike other microminerals (e.g., magnesium and iron), selenium crosses the hemochorial placenta via passive diffusion according to concentration gradient [66]. Animal studies have previously described that the fetus obtains Se from the maternal source in varying amounts, depending on the mother’s condition. However, in ruminants, the transfer of this mineral to the fetus occurs even when the maternal supply is low. These observations suggest that mothers may compromise their nutritional status to maintain fetal Se levels [67]. Research on camels has also revealed a higher concentration of Se in fetal vessels compared to maternal vessels, indicating a similar physiological behavior [68]. Se deficiency is directly associated with impaired fetal growth, mainly through the development of hypothyroid conditions [69]. In humans, reduced Se levels during pregnancy lead to increased oxidative stress, contributing to preterm labor, miscarriage, preeclampsia, and intrauterine growth retardation [66]. Uncontrolled ROS cause substantial cellular damage, representing the main mechanism of complication during pregnancy [10,49,70].

In the present study we demonstrate the immunoexpression of selenoproteins P, N, GPx-3, and Dio-3 in some chorionic villi blood capillaries and their contents. These villi present short projections, suggesting that they are sites of nutrient exchange in contact with the areolas of the maternal uterine glands [71]. Some studies have demonstrated the association between low blood Se levels and the incidence of fetal growth restriction, likely related to the effect of selenium on placental selenoprotein expression [70,71]. Se-P has been suggested as a reliable biochemical marker of Se status in humans and animals [30,72]. However, it is unlikely that there is a single indicator that can fully represent functional Se status; instead, a combination of markers is needed to reflect the specific issues associated with the suboptimal status of this mineral [73]. Se-P also plays a role in the transplacental transport of selenium to the fetus during late human pregnancy. Along with GPx-3, which is released into both fetal and maternal circulation, it is crucial for Se transport and acts as an antioxidant agent, protecting endothelial cells from peroxynitrite attack [71,74,75,76]. Studies on mouse placentas have shown that the Se-P gene is expressed during the last third of pregnancy, reaching maximum levels at term [77]. In addition to Se-P, the detection of GPx-3 at the maternal–fetal interface indicates that the fetus receives antioxidants. These antioxidants protect fetal tissues by reducing ROS levels during cellular metabolism and by regulating the cellular redox state during pregnancy [71,77,78,79]. The presence of Se-N was also demonstrated in the chorionic blood capillaries of the alpaca.

The presence of Dio-3 in the placenta is crucial for the metabolism of thyroid hormones, as it plays a role in the normal activity of the thyroid gland in converting thyroxine (T4) into its active form, triiodothyronine (T3) [69]. In humans, the regulation of these thyroid hormones by Dio-3 is essential for healthy fetal development. Dio-3 activity also increases with advanced gestational age in human placental cells [80,81,82]. Nutrient demand and oxidative stress activity increase during pregnancy. Micronutrient deficiencies, including Se, impair the antioxidant response, exposing the placenta to ROS accumulation and oxidative stress. In humans, maternal Se deficiency can lead to placental insufficiency and dysfunction, resulting in pregnancy complications such as pre-eclampsia, premature birth, gestational diabetes, and fetal growth restriction [34,71,82]. The detection and semiquantification of selenoproteins provides valuable information on physiological selenium status [77]. Therefore, a deeper understanding of placental selenoproteins could offer significant insights into the influence of selenium on the health of both mother and fetus [71].

## 5. Conclusions

Our results reveal morphological features and the immunoexpression of selenoproteins P, N, GPx-3, and Dio-3 in the epitheliochorial placenta of the alpaca. The characteristics of blood vessel localization and the abundance of collagen types in the chorion and amnion could be related to environmental characteristics. It is possible that the subepithelial blood capillaries maximize the diffusion of oxygen from the mother to the fetus and waste products in the reverse direction, optimizing the availability of this gas, which is in a low concentration in the alpaca’s environment. Variations in the type of collagen predominant in each of the annexes could be modulated by hypoxic conditions, although this needs to be demonstrated, along with the potential role of this fibrillar component in each annex. The immunoexpression of selenoproteins in the blood vessels of the chorion and amnion could be related to the antioxidant effect of these molecules on the fetus and placenta. There are no previous reports analyzing transplacental Se transport in SACs, so these preliminary results in alpacas could initiate future functional and comparative studies. This study has important implications for understanding placentation in South American camelids, as these animals can maintain prolonged pregnancies and ensure fetal survival and growth despite food restrictions and low oxygen availability in their environment.

## Figures and Tables

**Table 1:** Primary antibodies, dilution, and positive control.

Primary Antibody	Dates	Dilution	Positive Control
Selenoprotein P (B-9)	sc-376858. Mouse; monoclonal	1:50	Rat pancreas
Selenoprotein N (A-11)	sc-365824. Mouse; monoclonal	1:50	Human placenta
GPx-3 (23B1)	sc-58361. Mouse; monoclonal	1:50	Rat kidney
Dio-3	Merck Cat. ABS1073 Rabbit; polyclonal	1:100	Human placenta

Abbreviations: Dio-3, iodothyronine deiodinase; GPx-3, glutathione peroxidase.

## Data Availability

The original contributions presented in this study are included in the article. Further inquiries can be directed to the corresponding author.
